# Effects of Fermented Soybean Hulls on Reproductive Performance, Blood Physiology and Immune Parameters Along with Fecal Microbiota in Sows

**DOI:** 10.3390/ani14233389

**Published:** 2024-11-25

**Authors:** Xiuguo Shang, Yingjie Cui, Chaoyue Shang, Kaiguo Gao, Yujuan Chen, Yaodong Quan

**Affiliations:** 1 Department of Animal Science, College of Animal Science and Technology, Foshan University, Foshan 528225, China; xiuguoshang@163.com (X.S.); 15523290047@163.com (Y.C.); chenyujuan922@163.com (Y.C.); quanyaodong7826@163.com (Y.Q.); 2 College of Animal Science and Technology, Northwest Agriculture and Forestry University, Yangling 712100, China; chaoyueshang1028@163.com; 3 Institute of Animal Science, Guangdong Academy of Agricultural Sciences, Guangzhou 510640, China

**Keywords:** fermented soybean hulls, pregnant sows, reproductive performance, plasma biochemical indices, immunity, fecal flora composition

## Abstract

This study aims to investigate the effects of fermented soybean hulls (FSHS) on reproductive performance, plasma biochemical profiles, immune parameters, and fecal microbiota in sows. A total of 325 sows were randomized into five groups, each fed a diet with varying FSHS proportions: 0% (control), 2% (FSHS2), 4% (FSHS4), 6% (FSHS6), and 8% (FSHS8). Diets were prepared by replacing conventional soybean hulls with FSHS in equal amounts. The study lasted from day 30 to day 110 of gestation. The results indicated the following: (1) Reproductive Performance: The number of live piglets per litter, newborn litter weight, and individual piglet weight increased linearly with the increase in FSHS addition in the diet (P_Linear_ < 0.05). A trend toward reduced farrowing duration was observed when FSHS was added to the diet (P_Linear_ = 0.076). FSHS improved newborn weight and weight variation compared to the control when FSHS was added to the diet (P_Anova_ < 0.05). Replacing soybean hulls with FSHS also increased fecal scores, enhancing fecal consistency and alleviating constipation (*p* < 0.01). (2) Plasma Biochemical Indicators: Plasma urea concentration on day 60 decreased linearly with increasing FSHS (P_Linear_ < 0.05). Plasma total protein and aspartate aminotransferase (AST) activities showed quadratic effects (P_Quadratic_ < 0.05) on day 60 and 90 of gestation, while alkaline phosphatase activity on day 90 showed a quadratic trend (P_Quadratic_ = 0.082). (3) Hormone Levels: Plasma insulin and adrenaline levels exhibited quadratic effects on day 60 and 90 of gestation, respectively (P_Quadratic_ < 0.05). (4) Immune Parameters: Plasma immunoglobulin G (IgG) and M (IgM) levels increased linearly with FSHS proportion on day 60 (P_Linear_ < 0.05), but no significant changes were observed on day 90 (*p* > 0.05). (5) Fecal Microbiota: On day 90, the abundance of actinobacteriota increased quadratically with FSHS levels (P_Quadratic_ < 0.05), while bacteroidota decreased linearly (P_Linear_ < 0.05). During parturition, actinobacteriota abundance continued to increase linearly (P_Linear_ < 0.05). Notably, the FSHS8 group had higher actinobacteriota abundance compared to the control group (*p* = 0.05). At the genus level, terrisporobacter showed a quadratic increase and then decrease (P_Quadratic_ < 0.05), while ruminococcus showed a quadratic decrease and then increased (P_Quadratic_ < 0.05). Both terrisporobacter in the FSHS2 and FSHS6 groups and ruminococcus in the FSHS8 group were more abundant compared to the control (*p* < 0.01). Conclusions: Dietary supplementation with FSHS increased fecal microbial diversity, alleviated the incidence of constipation, and ultimately improved sow reproductive performance. The recommended optimal addition of FSHS to the sow diet is 6%.

## Simple Summary

Dietary fiber has a number of benefits for gestating sows, including enhancing satiety, regulating backfat thickness, promoting intestinal motility, and improving intestinal health and physiological metabolism. Soybean hulls are a common ingredient for supplemental fiber in swine feed. However, the utilization of soybean hulls by sows is inefficient due to the digestive characteristics of monogastric animals, which results in a waste of feed resources. Pretreatment of cellulose from soybean hulls using fermentation technology is important for improving the nutritional efficacy of fiber in swine diets. This study aims to evaluate the effect of fermented soybean hulls (FSHS) on reproductive performance, plasma biochemical profiles, immune parameters, and fecal microbiota in sows. Dietary supplementation with FSHS enhanced immune function, increased fecal microbial diversity, alleviated the incidence of constipation, and ultimately improved sow reproductive performance. The recommended optimal addition of FSHS to the sow diet is 6%.

## 1. Introduction

The reproductive performance of pregnant sows is influenced by various factors such as physical condition, backfat thickness, and intestinal health. Fiber nutrition strategies are commonly employed in sow diets to address these factors [1]. Research has demonstrated that dietary fiber can enhance satiety, regulate backfat thickness, promote intestinal peristalsis, improve intestinal health and physiological metabolism, and ultimately boost reproductive performance [2,3]. Despite the widespread use of fiber ingredients in sow diets, monogastric animals, including sows, lack cellulase in their digestive fluids and are unable to directly decompose cellulose. Consequently, the nutritional benefits of fiber are largely dependent on microbial fermentation in the hindgut [4]. However, due to the short retention time of chyme in the intestines of monogastric animals, microbial fermentation of fiber is less efficient compared to ruminants, leading to reduced utilization of dietary fiber. Studies have reported monogastric animals have an inherent disadvantage in utilizing fiber [5]; that is, monogastric animals have low fiber digestibility. Therefore, it is of great significance to use technical methods to pre-treat feed materials with high fiber content in order to improve the nutritional efficacy of fiber in pig diets.

Fermentation technology has been widely applied to pre-treat fiber-rich unconventional ingredients in order to improve their digestibility and utilization by monogastric animals [6,7,8]. Fermentation of various materials, such as cottonseed meal [9], beet pulp [10], rice bran [11], soybean hulls [12], traditional Chinese medicine residues [13], and fermentation by-products [14], has been shown to enhance intestinal health. However, these studies typically involved conventional microbial fermentation processes. Microbial enzyme synergistic fermentation technology, known for its efficacy in processing protein ingredients, had principles particularly suited for enhancing the nutritional value of fiber ingredients. Whereas high-quality fiber ingredients such as soybean hulls, alfalfa meal, and beet pulp have frequently been used in sow diets, the use of lactic acid bacteria for anaerobic fermentation of these materials has been shown to improve reproductive performance [15]. In spite of this result, there is limited literature on the application of microbial-enzyme synergistic fermentation technology to fiber ingredients, and reports on the effects of such treatments on sow reproductive performance and physiological metabolism have not been found.

This study applied microbial-enzyme synergistic fermentation technology to pre-treat soybean hulls and substituted conventional soybean hulls with fermented soybean hulls (FSHS) in sow diets. The impact of FSHS on reproductive performance, plasma biochemical parameters, and immune indices, as well as fecal flora composition in pregnant sows, was investigated. Moreover, this research aims to provide technical and theoretical insights into the effective development and utilization of fiber ingredients in enhancing sow reproductive performance.

## 2. Materials and Methods

### 2.1. Production Process of FSHS

FSHS production process was provided by Yisheng (Yangjiang) Biology Technology Co., Ltd., Guangdong Province, China. Firstly, the dried soybean hulls were baked at 110 °C for 10 min and then ground to be sieved through a 40-mesh sieve. Next, they were mixed with sterile water, enzyme preparations (cellulase with an activity > 25,000 U/g), and inoculated with microorganisms (1 × 10^7^ CFU/g Aspergillus oryzae activated spores, 1 × 10^9^ CFU/g yeast and bacillus subtilis mixed culture). After mixing, the moisture content of the mixture is 50%. Afterward, these mixtures were placed in 1 m^3^ wooden boxes for fermentation. The mixture was fermented in a clean environment at 32 °C for 5 days, and stirred and mixed again on the 3rd day of fermentation. Finally, the mixture was dried at approximately 45 °C, then ground and packaged for use. The nutritional components of soybean hulls before and after fermentation are shown in Table 1. 

### 2.2. Experimental Design

All experimental procedures involved in this study were approved by the Animal Ethics Committee of Foshan University (Foshan, China). The experiment employed a single-factor randomized block design. A total of 325 sows (Large White × Landrace), characterized by similar body weights, consistent genetic backgrounds, optimal body conditions, and comparable estrus and breeding dates, were selected for the study. These sows, ranging from 3 to 6 parities, were randomly divided into five groups, with each group consisting of 65 replicates and each replicate containing one sow. The basal diet was formulated according to the NRC (2012) and China Pig Nutrition Requirements (2020), with its composition and nutritional levels shown in Table 2. In the experimental groups, the FSHS was gradually increased and used to replace an equivalent amount of conventional soybean hulls in the basal diet. The proportions of FSHS in each group were 0% (FSHS0 group, control), 2% (FSHS2 group), 4% (FSHS4 group), 6% (FSHS6 group), and 8% (FSHS8 group), corresponding to conventional soybean hull proportions of 8%, 6%, 4%, 2%, and 0%, respectively.

### 2.3. Feeding Management

The experiment was conducted at a large commercial pig farm in Guangxi Province, China. Sows naturally came into estrus and were artificially inseminated (the boar semen used was from Duroc boars with consistent genetic backgrounds and diluted semen from the same batch). Pregnancy status was determined by B-ultrasound from the 19th to the 21st day after post-breeding. Sows confirmed pregnant were fed the experimental diets from the 30th day of pregnancy, marking the beginning of the formal experiment. Sows were limited to feeding 2.5 kg/day during early gestation (day 30 to 90), and 3.0 kg/day during late gestation (day 90 to 110). The experimental diet was fed twice a day (08:00 and 16:00). Sows were housed individually with free access to water and moved to the farrowing house at day 110 of gestation. During the period from the 10th day of pregnancy to delivery, according to NRC (2012) and China Pig Nutrition Requirements (2020), routine feeding was carried out from the 10th day of pregnancy to delivery. Other management practices follow the standard management procedures and regular immunization programs of the farm. Sows that aborted or had other conditions affecting the experimental results were removed from the study.

### 2.4. Blood Samples Collection and Determination

On the 60th and 90th day of pregnancy, 5 mL blood was collected from the ear vein of six randomly selected sows in each group two hours after morning feeding. Blood samples were collected in heparin sodium tubes and centrifuged at 2500 r/min and 4 °C for 20 min to obtain plasma. Then, these plasma samples were equally divided into 1.5 mL EP tubes and stored at −20 °C. Backfat thickness at point P2 (the outer tangent of the last rib of the pig is 6.5 cm from the dorsal midline) was measured using an ultrasound on the 30th and 110th day of pregnancy. During farrowing, the total number of piglets born, number of live piglets, number of piglets lost, litter birth weight, individual piglet birth weight, and duration of farrowing were recorded. Piglets weighing more than 1000 g with pink skin and strong vitality at birth were considered healthy.

Biochemical indices in plasma, including total protein, albumin, urea, glucose, triglycerides, cholesterol, high-density lipoprotein, low-density lipoprotein, calcium, phosphorus levels, and the activities of aspartate aminotransferase, alanine aminotransferase, alkaline phosphatase, and lactate dehydrogenase, were measured using an automatic biochemical analyzer (SELECTRA ProXL, VITAL). Hormone and immune indices in the plasma were determined using enzyme-linked immunosorbent assay (ELISA) kits from Shanghai Enzyme-linked Biotechnology Co., Ltd. (Shanghai, China) and Zhong sheng Bei kong biotechnology Co., Ltd. (Beijing, China). The information on the reagent kits used in this study is shown in Table S1.

### 2.5. Fecal Determination

On the day of delivery, the feces of each group of sows who were defecating were checked and photographed, and the feces were scored according to the feces scoring standard. 0: No feces; 1: dry and granular; 2: between dry and normal feces; 3. The feces are normal, soft, firm, and well-shaped; 4. It is between normal feces and thin feces, with a stylish appearance, but not firm; 5: Very wet feces, unformed and liquid.

### 2.6. Fecal Microbiota Determination

Three sows in each group were randomly selected for fecal sample collection. Feces were collected on the 60th and 90th day of pregnancy and on the morning of parturition. Fecal samples are collected from fresh feces and immediately stored in sampling bags to avoid any environmental pollution. Then, these samples were temporarily stored in liquid nitrogen until measurement.

Fecal samples from the three gestational periods were subjected to 16S rRNA gene sequencing. The following procedures were performed: DNA Extraction from Feces Microbial DNA was extracted from the fecal samples using a DNA extraction kit, following the protocol provided by the manufacturer. After extraction, the DNA concentration and integrity were assessed using a spectrophotometer and agarose gel electrophoresis. The DNA samples that met the quality criteria proceeded to the next step. 16S rRNA Amplification and Sequencing-specific primers with barcodes were used according to the sequencing regions. The primers for the 16S V4 region were 515F-806R; for the 18S V4 region, 528F-706R; for the 18S V9 region, 1380F-1510R; for the ITS1 region, ITS5-1737F and ITS2-2043R; and for the ITS2 region, ITS3-2024F and ITS4-2409R. After amplification, the integrity of the products was verified by agarose gel electrophoresis. Gel recovery kits were used to purify the products. Sequencing was performed by Novogene Bioinformatics Technology Co., Ltd. on the Illumina MiSeq sequencing platform. Library construction was carried out using the NEB Next^®^ Ultra™ DNA Library Prep Kit for Illumina from New England Biolabs. Following quantification by Qubit and library quality assessment, sequencing was conducted using the HiSeq platform.

### 2.7. Data Statistics and Analysis

Experimental data were analyzed using SPSS version 25.0 for one-way analysis of variance (ANOVA). Multiple comparisons were performed using the Tukey test to identify significant differences between experimental groups. The general linear model was employed to assess both linear and quadratic effects of the experimental treatment factors, specifically the FSHS addition ratio, on sows’ reproductive performance. Data are presented as means ± standard error of the mean (SEM). A significance level of *p* ≤ 0.05 was considered statistically significant, while 0.05 < *p* < 0.10 was considered indicative of a trend.

## 3. Results

### 3.1. Effects of FSHS on Reproductive Performance of Sows

As detailed in Table 3, increasing the FSHS ratio in the experimental diet led to a linear increase in the number of healthy piglets per litter, newborn litter weight, and individual piglet weight (P_Linear_ < 0.05), while the standard deviation of newborn weight decreased linearly (P_Linear_ < 0.05). There was also a trend toward a linear reduction in far-rowing duration (P_Linear_ = 0.076). The FSHS supplementation improved newborn weight and the standard deviation of individual newborn weight (P_ANOVA_ < 0.05). Compared to the control group (FSHS0), the high-dose groups (FSHS6 and FSHS8) showed an increase in newborn weight (P_ANOVA_ < 0.05). Additionally, the standard deviation of individual newborn weight in the FSHS4, FSHS6, and FSHS8 groups was lower than that in the control and FSHS2 groups (P_ANOVA_ < 0.05).

### 3.2. Changes in Fecal Consistency at Parturition in Pregnant Sows Fed with FSHS

**Figure 1:**
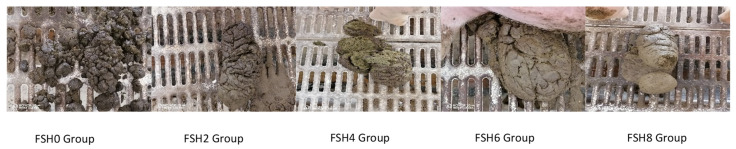
Effects of FSHS on sow fecal morphology.

As illustrated in Table 4 and Figure 1, throughout the experimental period, the fecal scores indicated that the fecal scores of the experimental groups were higher than those of the control group (*p* < 0.01). The fecal scores of the sows in the control group at parturition ranged from 1 to 2, whereas the scores for the remaining experimental groups (FSHS2, FSHS4, FSHS6, and FSHS8) were between 2 and 4. The substitution of soybean hulls with FSHS in the diet visibly improved the consistency of the feces and alleviated the incidence of constipation in the sows.

### 3.3. Effects of FSHS on Plasma Biochemical Indices of Pregnant Sows

As indicated in Table 5, increasing the proportion of FSHS in the experimental diet resulted in a linear decrease in plasma urea content on day 60 of gestation (P_Linear_ < 0.05). Plasma total protein content, aspartate aminotransferase, and lactate dehydrogenase activity showed quadratic effects on day 60 and 90 of gestation (P_Quadratic_ < 0.05). On day 90, plasma alkaline phosphatase activity exhibited a trend toward a quadratic effect (P_Quadratic_ = 0.082). On day 60, plasma aspartate aminotransferase activity was higher in the FSHS8 group compared to the FSHS2, FSHS4, and FSHS6 groups (P_ANOVA_ < 0.05). On day 90, aspartate aminotransferase activity was higher in the FSHS4 group than in the FSHS2 and FSHS8 groups (P_ANOVA_ < 0.05). Additionally, plasma calcium and phosphorus concentrations decreased linearly with increased FSHS levels on day 60 of gestation (P_ANOVA_ < 0.05), but this trend was not observed on day 90.

### 3.4. Effects of FSHS on Plasma Hormone Levels in Pregnant Sows

As shown in Table 6, increasing the FSHS proportion in the experimental diet resulted in a quadratic effect on plasma insulin levels on day 60 of gestation, which initially decreased and then increased (P_Quadratic_ < 0.05). Plasma epinephrine levels on day 90 also exhibited a quadratic effect (P_Quadratic_ < 0.05). On day 60, the FSHS8 group had higher plasma insulin levels compared to the control group (P_ANOVA_ < 0.05). On day 90, plasma adrenaline levels in the FSHS2 group were lower than those in the control group (P_ANOVA_ < 0.05). No significant differences were observed among treatment groups for progesterone, estradiol, cortisol, growth hormone, insulin-like growth factor I, leptin, and ghrelin levels on day 60 or 90 of gestation.

### 3.5. Effects of FSHS on Plasma Immunoglobulin Levels in Pregnant Sows

As shown in Table 7, with the gradual increase in the proportion of FSHS in the experimental diet, the plasma levels of immunoglobulin G (IgG) and immunoglobulin M (IgM) on day 60 of gestation increased linearly (P_Linear_ < 0.05), whereas there was no significant effect on day 90 of gestation (*p* > 0.05). On day 60 of gestation, the plasma IgM levels in the FSHS8 group were higher than those in the control group (P_ANOVA_ < 0.05).

### 3.6. Impact of FSHS on the Microbial Community in the Feces of Pregnant Sows

#### 3.6.1. Assessment of Microbial DNA Quality in Fecal Samples

**Figure 2:**
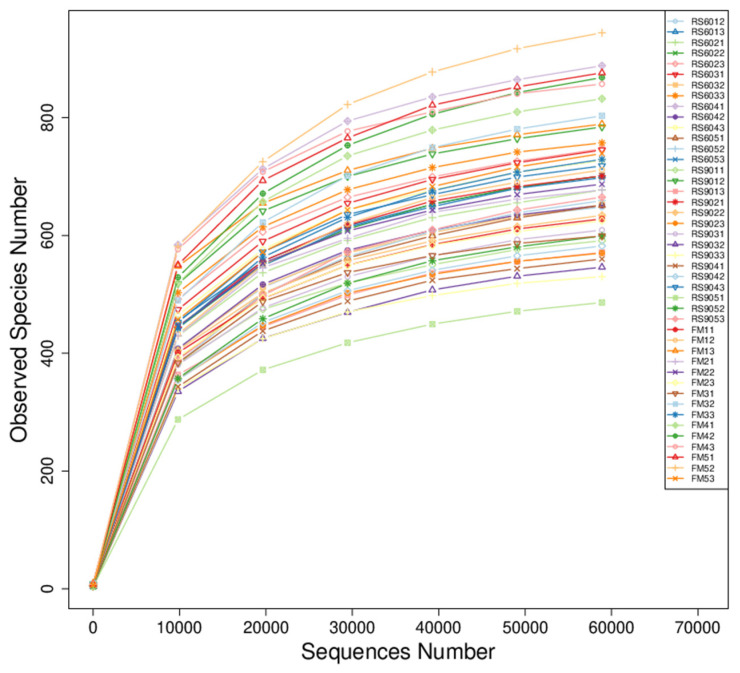
Sow fecal dilution curve. Dilution curve is used to compare the richness of sample species with different sequencing numbers. The abscissa position of the extended end point of the sample curve is the sequencing number of the sample.

High-throughput microbial isolation and cultivation techniques yielded 62.9% of bacterial taxa present in sow feces. As depicted in Figure 2, an increase in sample size led to the saturation of bacterial taxa within the fecal microbial community, indicating that the microbial diversity represented encompasses the majority of microbial taxa found in each individual sow’s fecal matter.

**Figure 3:**
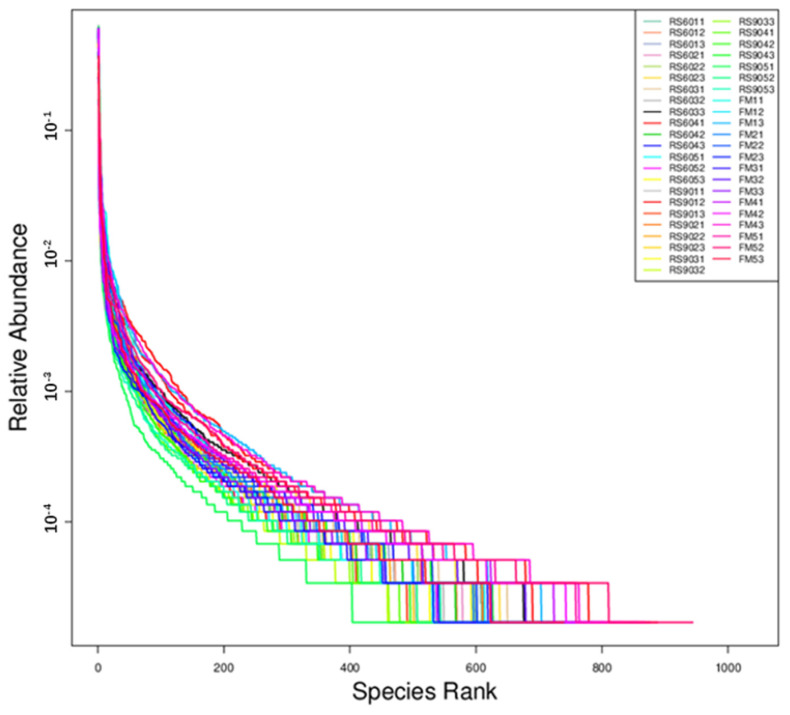
Grade abundance curve. The curve arranges each sample in sequence according to its abundance along the abscissa, and connects OTU/ASV with each other by a broken line or curve with their respective abundance values as the ordinate, thus reflecting the distribution law of OTU abundance in each sample.

Figure 3 illustrated that, on the horizontal axis, the broad span of the curve suggested a high richness of fecal microbiota. On the vertical axis, the relatively smooth curve indicated a uniform distribution of microorganisms within the feces, enhancing the reliability of the results. The rank–abundance curve constructed provides a direct reflection of both the richness and evenness of the microbial communities in the feces of the sows.

**Figure 4:**
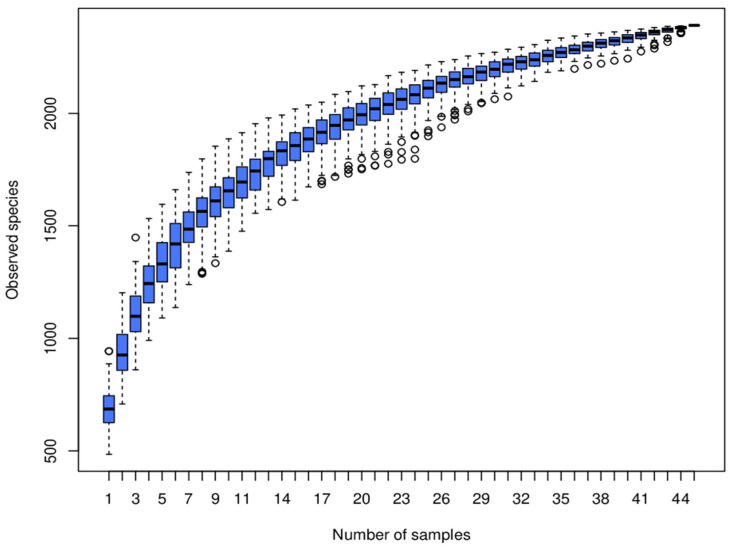
Species accumulation box diagram. The *y*-axis represents species richness, and the x-axis represents samples. The circle represents the data points beyond the normal range. The blue box in the figure represents the distribution interval of the middle 50% of this group of data, and the middle line is the median.

As shown in Figure 4, an increase in fecal sample quantity corresponded to a rise in the number of Operational Taxonomic Units (OTUs). The curve in the boxplot stabilized as the number of OTUs approached saturation, indicating that the addition of new OTUs became increasingly rare. This suggested that the sequencing depth achieved in this study provides an accurate representation of the microbial community composition in sow feces.

#### 3.6.2. Analysis of Sample Complexity

According to the results presented in Table 8, no significant differences in microbial diversity and richness were observed in the feces of pregnant sows on day 60 and 90 of gestation (*p* > 0.05). The supplementation of the diet with FSHS as a replacement for soybean hulls did not impact the microbial diversity in the feces of sows at parturition. However, compared to the FSHS4 group, the FSHS8 group exhibited an increase in microbial richness (Chao1 index, *p* < 0.05; ACE index, *p* < 0.05). This indicates that the replacement of soybean hulls with FSHS in the diet resulted in an enhancement of microbial richness in the feces of sows at parturition. Nevertheless, no differences were noted in the Shannon index across various stages (*p* > 0.05), suggesting that the species diversity composition remained similar among all groups and time points.

#### 3.6.3. Diversity Comparison Analysis

**Figure 5:**
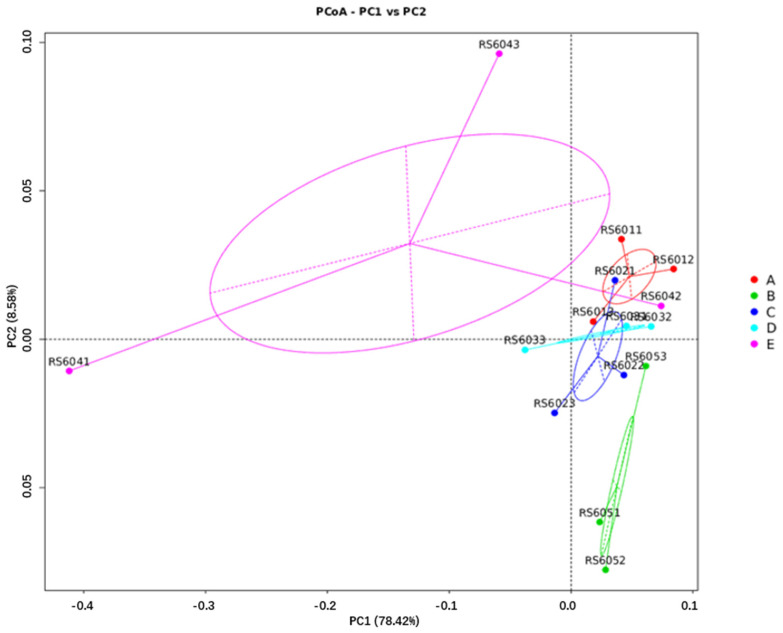
PCOA analysis of fecal flora at 60 d gestation.

**Figure 6:**
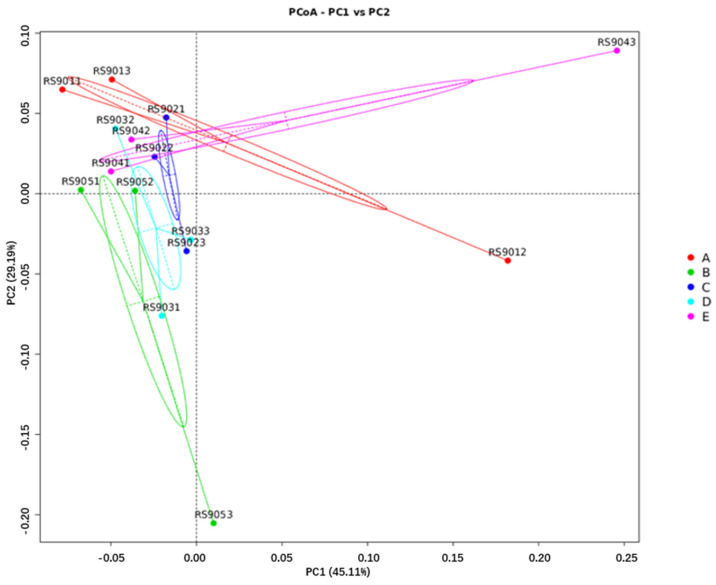
PCOA analysis of fecal flora at 90 d gestation.

**Figure 7:**
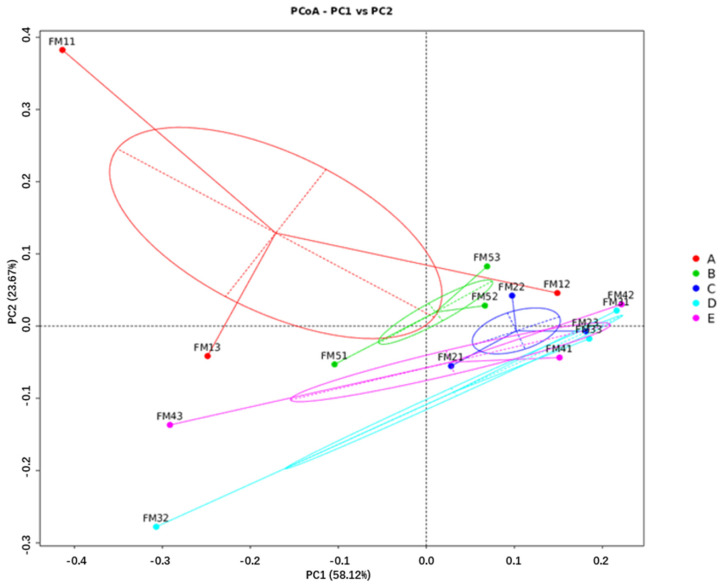
PCOA analysis of fecal flora at sow delivery.

Significance testing of community structure differences among groups revealed that no distinct clusters were formed on day 60, 90 of gestation, or at parturition. The levels of FSHS substitution did not have a significant impact on microbial community structure (ANOSIM test, *p* > 0.05). According to the PCoA plots (Figure 5, Figure 6 and Figure 7), on day 60 of gestation, the Beta diversity of the FSHS8 group was found to be higher than that of the FSHS0 group (Wilcoxon test, *p* < 0.05). No significant differences were observed at other time points (Wilcoxon test, *p* > 0.05).

### 3.7. Impact of FSHS on the Microbial Community Structure and Distribution in the Feces of Pregnant Sows

#### 3.7.1. Changes in Relative Abundance at the Phylum Level

As shown in Table 9, at the phylum level, there were no significant differences in the relative abundance of microbial communities in the feces of the experimental groups compared to the FSHS0 group on day 60 and 90 of gestation (*p* > 0.05). However, on day 90 of gestation, the abundance of actinobacteriota exhibited a quadratic increase followed by a decrease with increasing FSHS substitution (P_Quadratic_ < 0.05). At parturition, a linear decrease in the abundance of bacteroidota and a linear increase in the abundance of actinobacteriota were observed with increasing FSHS substitution (P_linear_ < 0.05). A trend was noted where the abundance of actinobacteriota in the FSHS8 group was higher compared to the FSHS0 group (*p* = 0.05).

#### 3.7.2. Changes in Relative Abundance at the Genus Level

According to Table 10, at the genus level, the abundance of terrisporobacter exhibited a quadratic increase followed by a decrease with increasing FSHS substitution on day 60 of gestation (P_Quadratic_ < 0.05), while the abundance of ruminococcus UCG-002 showed a quadratic decrease followed by an increase (P_Quadratic_ < 0.05). The abundance of parabacteroides was higher in the FSHS2 group compared to the FSHS8 group (*p* < 0.05). The abundance of terrisporobacter was higher in the FSHS2 and FSHS6 groups compared to the FSHS0 group (*p* < 0.01). The abundance of ruminococcus UCG-002 was higher in the FSHS8 group compared to all other experimental groups and the FSHS0 group (*p* < 0.01). No significant differences in the abundance of these genera were observed among the groups on day 90 of gestation and at parturition (*p* > 0.05).

## 4. Discussion

### 4.1. Effects of FSHS on Reproductive Performance in Pregnant Sows

Fermented feed can degrade raw materials into bio-fermented feed containing microbial cell proteins, amino acids, probiotics, and compound enzyme preparations by using microorganisms and other substances as a starter, which can effectively improve the intake of animal nutrients, improve the quality and taste of feed, and improve the production performance of poultry.

The reproductive performance of sows mainly includes the number of healthy offspring, birth weight, birth litter weight, and birth weight variation. Adding an appropriate amount of dietary fiber could increase the number of piglets per litter. For instance, including 0.6% konjac flour in the diet improved both the total number of piglets per litter and the number of live piglets by 0.6 and 0.4, respectively, compared to the control group [16]. Fermented soybean meal (FSBM) has also been shown to improve animal performance [17]. These studies support the idea that adding appropriate fiber to the gestation diet can enhance sow reproductive performance. Our findings align with these results, demonstrating that the fiber nutritional benefits of FSHS positively impact the number of live piglets. However, the effect and influence degree of different fiber types are quite different.

Currently, there is limited literature on the effects of enzyme-assisted fermentation technology on dietary fiber and sows’ reproductive performance. However, studies indicated that adding lactic acid bacteria-fermented alfalfa powder to gestation diets improved piglet uniformity and reduced intrauterine growth retardation, which positively affects sow reproductive performance [18]. Although anaerobic fermentation differs from the technology used in this study, FSHS retained essential nutritional characteristics from fermentation. This improved sow body condition and reproductive performance, notably increasing backfat thickness, number of piglets per litter, number of live piglets, and birth weight [19].

Our study found a linear increase in sow backfat thickness with higher FSHS proportions in the diet. This effect might derive from the breakdown of fiber in FSHS into digestible monosaccharides and oligosaccharides, which reduced the anti-nutritional factors in soybean hulls and enhanced fermentation-active factors, proteins, peptides, and soluble amino acids. This improved nutrient digestion and absorption, benefiting backfat accumulation and reproductive performance, consistent with reports on the nutritional effects of fermented feed [19]. Additionally, feeding FSHS to Wulong geese, which contained amino acids, polysaccharides, and bioactive peptides, improved nutrient utilization efficiency and protein synthesis without altering feed intake [20].

One possible reason is that the anti-nutritional factors of soybean skin, such as trypsin inhibitor and antigen protein, are reduced during microbial fermentation, which improves the nutritional value of soybean skin. Lin Yuan et al. determined the parameters of soybean peptides and anti-nutritional factors in FSBM, and further piglet-feeding experiments showed that the average daily gain (ADG) and feed conversion rate (FCR) of piglets fed with 10% FSBM were higher than those fed without FSBM (*p* < 0.05), indicating that FSBM had a positive effect on the nutrient digestibility of piglets [21]. Previous research has shown that pigs fed a diet containing FSBM had a greater ADG and FCR than the control group [22,23,24]. FSBM can reduce the diarrhea rate and mortality rate of suckling piglets, which may be due to the existence of beneficial microorganisms inhibiting the proliferation of pathogenic microorganisms [25] and microbial fermentation reducing or removing antigen proteins [26]. Another potential mechanism was that FSHS enhanced gut microbial diversity and abundance. Beneficial gut bacteria more effectively utilize pre-treated FSHS substrates, promoting the growth of beneficial microorganisms and maintaining microbial balance [27]. Increased levels of beneficial bacteria led to higher production of volatile fatty acids, which lowered gut pH and inhibited harmful pathogens [28]. This improved gut health and overall sow health, thereby enhancing reproductive performance [29]. Further research was needed to confirm the effects of FSHS on gut microbial abundance and diversity in sows.

### 4.2. Effects of FSHS on Plasma Biochemical Indicators in Pregnant Sows

Research has shown that fermenting feed ingredients enhanced amino acid content, reduced anti-nutritional factors, and improved nutritional quality and utilization efficiency, as a result, benefiting animal health and metabolic function [30]. This study used plasma biochemical indicators to assess the impact of FSHS on nutrient digestion and absorption in sows, providing direct evidence of how the fermented material affected sow condition and embryo development [31,32].

Results showed that plasma total protein and aspartate aminotransferase (AST) activity exhibited a quadratic effect. This may be due to FSHS’s influence on liver physiology and metabolism. Research has shown that fermented soybean paste attenuates liver damage in obese mice [33]. AST activity was generally higher on day 60 of gestation compared to day 90, reflecting the varying nutritional demands of the embryos, mammary glands, and sow at different pregnancy stages, which affected liver health and metabolism [34,35]. Notably, plasma AST activity and total protein content in the FSHS6 group were near the peak of the quadratic effect, suggesting that 6% FSHS most positively impacts liver function. This is further supported by growth data showing the smallest standard deviation in piglet birth weight in the FSHS6 group, indicating that 6% FSHS enhances physiological metabolism, liver protein, and amino acid metabolism, and promotes balanced embryo development and piglet uniformity.

On day 60 of pregnancy, FSHS led to a linear decrease in plasma urea levels, indicating improved efficiency in protein and amino acid utilization [36]. This reduction in urea nitrogen reflects better utilization of dietary nitrogen by the sow and fetus and provides direct evidence of increased piglet birth weight. Studies have shown that during pregnancy, maternal circulating levels of calcium increase in order to support healthy skeletal development [37,38]. In this study, plasma alkaline phosphatase activity demonstrated a quadratic effect under FSHS influence, with 6% FSHS reaching the peak, indicating that 6% FSHS benefits calcium and phosphorus metabolism and fetal development. The specific mechanisms of this effect warrant further investigation.

### 4.3. Effects of FSHS on Hormone Levels in Pregnant Sows’ Plasma

Previous research has demonstrated that fiber can influence sow hormone secretion, thereby impacting fetal development [39]. For instance, betaine addition in both sow and sow-offspring diets influences the plasma hormones related to ingestion [40]. Sun Zhonghua et al. found that the contents of Glu, TG, leptin, and insulin in the serum of piglets were significantly decreased [41], while leptin and insulin both inhibited appetites, and the changes in the related indexes of serum were consistent with the phenotype of increased appetite of piglets. In this study, plasma adrenaline levels on day 90 of pregnancy displayed a quadratic effect, though no direct comparisons are available in the literature. Adrenaline’s role in energy metabolism suggests that an appropriate FSHS ratio might help sows store energy and facilitate metabolic processes during pregnancy [42].

Insulin, a critical hormone for glucose metabolism, also exhibited a quadratic effect on day 60 of pregnancy with FSHS supplementation. Fiber affects not only insulin secretion but also insulin receptor sensitivity, helping to regulate and stabilize blood glucose levels [43,44]. Pregnant sows often face challenges such as gestational diabetes and insulin resistance [45]. Post-fermentation, FSHS’s nutritional profile extends beyond fiber, encompassing monosaccharides, oligosaccharides, amino acids, peptides, and microbial metabolites, all of which influence glucose metabolism and insulin levels [46].

Whereas FSHS appears to impact insulin levels and glucose metabolism positively, further research is needed to elucidate the specific mechanisms underlying these effects.

### 4.4. Effects of FSHS on Immunoglobulin Levels in Pregnant Sows’ Plasma

Fermented feed ingredients have been shown to boost animal immunity. For instance, feeding FSBM to sows has been reported to increase immunoglobulin A (IgA) and immunoglobulin M (IgM) levels in colostrum [47]. Similarly, other studies have observed improvements in IgA, IgM, and immunoglobulin G (IgG) levels in sow serum following the fermentation of corn [48]. A notable increase in serum IgG levels was also reported in pigs after fermenting a mixture of pomegranate, ginkgo, and licorice [49].

In this study, the use of FSHS led to a linear increase in plasma IgM and IgG levels on day 60 of pregnancy. Specifically, the FSHS8 group exhibited significantly higher IgM levels compared to the control group. This suggests that FSHS, which included oligosaccharides, peptides, and microbial metabolites, positively influenced sow immunity. These findings align with previous research demonstrating the beneficial effects of fermented feed on immune function [49,50]. Enhanced sow immunity, in turn, supports fetal development and improves reproductive performance, which indirectly explains the increased number of live piglets observed in this study [51].

### 4.5. Impact of FSHS in Gestating Sow Diets on Fecal Microbiota Composition

Fecal microbiota, representing the microbial community in the hindgut, provides insights into microbial distribution within the intestinal tract. The swine hindgut hosts a diverse microbial population influenced by genetics, environmental factors, and diet. Dietary fiber served as a crucial substrate for microbial growth and survival [32]. Fiber interacts with the intestinal mucosa and resident microbiota, playing a role in regulating gut health. FSHS, when processed extracorporeally, bypass the lengthy microbial fermentation process in the intestine, offering a direct nutritional benefit.

Using bio-fermented feed can reduce the colonization of intestinal pathogenic bacteria in piglets and improve intestinal health [52]. Studies have shown that the use of bio-fermented feed can reduce the abundance of harmful bacteria in piglets’ intestines to a certain extent, which is beneficial to the health of piglets’ intestines [41]. At the same time, using fermented cottonseed meal instead of a soybean meal diet can also reduce the abundance of Geotrichum in the cecum and increase the abundance of Ruminococcus, thus improving the growth performance and antioxidant capacity of piglets [53].

In this study, the microbial community at the phylum level remained relatively stable from gestational day 60 to parturition. The predominant taxa were Proteobacteria, Bacteroidota, and Firmicutes, collectively comprising 83.3% to 97.4% of the total microbial community. These findings indicate minimal variation in microbial composition at this level during gestation.

At the genus level, significant fluctuations were observed among the top genera, including Ruminococcus, Parabacteroides, Terrisporobacter, and others. Pathogenic genera such as Streptococcus, Treponema, and Escherichia-Shigella were present at gestational day 60, with the highest diversity of harmful species observed. By parturition, the diversity of these pathogenic genera decreased, likely due to the extracorporeal fermentation of FSHS, which aids in further fermentation in the rectum and suppresses harmful bacteria, thereby supporting intestinal health.

Alpha diversity analysis revealed that substituting soybean hulls with FSHS significantly increased microbial richness in the rectal contents at parturition. Beta diversity analysis, using PCoA plots, showed a distinct separation between the FSHS groups and the control group (FSHS 0) on day 60 of gestation and parturition. This suggests that FSHS substitution influences microbial distribution and community composition in the intestine, highlighting its potential impact on gut health and microbial balance.

## 5. Conclusions

Fermented soybean hulls (FSHS) positively affect sows’ health and reproductive performance. FSHS improves plasma biochemical indicators and hormone levels, boosts plasma immunoglobulin content, and enhances reproductive outcomes. Replacing soybean hulls with FSHS increased microbial abundance in the rectum of pregnant sows, supported a balanced gut microbiota, and elevated the presence of beneficial genera. Key benefits observed include increased piglet birth weight, enhanced individual piglet birth weight, reduced variability in piglet birth weight, shortened farrowing duration, greater number of live piglets, and so on. Based on the research results, it is suggested that the common soybean hull in the sow diet should be replaced by 6% FSHS to achieve the best improvement in physiological metabolism and reproductive performance.

## Figures and Tables

**Table 1:** Nutrient Levels of Soybean Hulls Before and After Fermentation (air-dry basis, %).

Items	Soybean Hulls	FSHS
Moisture	11.65	11.04
Crude Protein	12.27	14.21
Crude Ash	5.88	9.00
Crude Fiber	39.78	17.60
Neutral Detergent Fiber	58.25	42.20
Acid Detergent Fiber	45.52	23.60
Calcium	0.54	0.59
Total Phosphorus	0.32	0.42
Gross Energy/(MJ/kg)	17.63	17.68
Moisture	11.65	11.04

Note: The above nutrition levels were measured values.

**Table 2:** Composition and Nutrient Levels of the Basal Diet (air-dry basis, %).

Items	Content
Ingredients	
Corn	68.23
Soybean Meal	12.00
Soybean Hulls	8.00
Alfalfa Meal	7.00
Lysine	0.30
Methionine	0.06
Threonine	0.21
Tryptophan	0.06
Valine	0.04
NaCl	0.10
Premix ^1^	4.00
Total	100.00
Nutrient levels ^2^	
Digestible Energy (MJ/kg)	13.32
Crude Protein	13.55
Calcium	0.81
Total Phosphorus	0.60
Available Phosphorus	0.41
Standardized Ileal Digestible Lysine	0.72
Standardized Ileal Digestible Methionine	0.24
Standardized Ileal Digestible Threonine	0.58
Crude Fiber	6.40
Neutral Detergent Fiber	13.91
Acid Detergent Fiber	8.30

(^1^) The premix provided the following per kg of the diet: VA 10 000 IU, VD3 3 500 IU, VE 88 IU, VK3 1 mg, VB1 2 mg, VB2 5.0 mg, VB6 3 mg, VB12 0.02 mg, folic acid 3.5 mg, D-pantothenic acid 11.5 mg, nicotinic acid 25 mg, biotin 0.4 mg, choline chloride 800 mg, antioxidant 100 mg, Ca(CaCO_3_ and CaHPO_3_) 6.5 g, P (CaHPO_3_) 2.9 g, Cu (CuSO_4_) 8 mg, Fe (FeSO_4_) 125 mg, Mn (MnSO_4_) 30 mg, Zn (ZnSO_4_) 90 mg, I (KI) 0.20 mg, Se (Na_2_SeO_3_) 0.25 mg. (^2^) Nutrient levels were calculated values, which were calculated according to Tables of Feed Composition and Nutritive in China (31rd edition).

**Table 3:** Effects of FSHS on Reproductive Performance of Sows.

Items	Groups						*p*-Value		
	FSHS0 (Control)	FSHS2	FSHS4	FSHS6	FSHS8	SEM	ANOVA	Linear	Quadratic
Numbers of sow	48	46	42	45	45				
Numbers of litter	12.34	12.72	13.08	13.15	13.00	0.18	0.89	0.14	0.36
Numbers of healthy litter	10.80	11.30	11.79	11.81	11.78	0.15	0.23	0.02	0.22
Numbers of piglet loss	1.43	1.23	1.27	1.25	1.02	0.10	0.88	0.26	0.90
Newborn litter weight, kg	15.40 ^b^	16.42 ^ab^	16.95 ^ab^	17.14 ^a^	17.31 ^a^	0.19	0.04	0.00	0.20
Newborn weight, kg	1.27	1.29	1.31	1.32	1.34	0.01	0.63	0.01	0.87
Newborn weight variation, kg	0.31 ^a^	0.27 ^a^	0.23 ^b^	0.17 ^c^	0.24 ^b^	0.01	0.02	0.00	0.21
Backfat thickness on the 30th day of pregnancy, mm	15.17	15.13	15.21	15.16	15.16	0.19	0.99	1.00	0.96
Backfat thickness on the 110th day of pregnancy, mm	17.39	18.30	18.15	18.65	18.06	0.30	0.65	0.39	0.36
Backfat thickness gain, mm	2.78	3.22	3.26	3.65	3.41	0.12	0.48	0.03	0.30
Labor time, min	236.90	222.71	210.03	207.47	207.59	5.46	0.70	0.08	0.39

Note: In the same row, values with no letter or the same letter superscripts mean no significant difference (*p* > 0.05), while with different small letter superscripts mean significant difference (*p* < 0.05).

**Table 4:** Fecal scores of parturition sows.

Items	Control Group	Experimental Group	SEM	*p*-Value
Fecal score	1.17 ^b^	3.00 ^a^	0.143	<0.001

Note: Fecal scoring standard: 0: No feces; 1: dry and granular; 2: between dry and normal feces; 3. The feces are normal, soft, firm, and well-shaped; 4. It is between normal feces and thin feces, with a stylish appearance, but not firm; 5: Very wet feces, unformed and liquid. In the same row, values with no letter or the same letter superscripts mean no significant difference (*p* > 0.05), while with different small letter superscripts mean significant difference (*p* < 0.05).

**Table 5:** Effects of FSHS on Plasma Biochemical Indices of Pregnant Sows (*n* = 30).

Items	Groups						*p*-Value		
	FSHS0 (Control)	FSHS2	FSHS4	FSHS6	FSHS8	SEM	ANOVA	Linear	Quadratic
The 60th day of pregnancy									
Total Protein, g/L	87.79	83.66	80.55	83.00	84.61	0.857	0.099	0.223	0.017
Alanine Aminotransferase, U/L	63.28	47.98	55.87	53.63	57.23	2.391	0.381	0.704	0.176
Aspartate Aminotransferase, U/L	46.75 ^ab^	35.78 ^b^	36.61 ^b^	33.75 ^b^	62.22 ^a^	2.773	0.001	0.063	0.001
Alkaline Phosphatase, U/L	57.13	59.06	73.36	58.33	74.80	6.222	0.834	0.459	0.996
Lactic dehydrogenase, U/L	447.60	396.52	421.87	412.99	496.20	18.773	0.514	0.405	0.153
Albumin, g/L	40.21	33.72	35.89	38.52	37.44	0.891	0.180	0.904	0.125
Urea, mmol/L	3.80	3.61	3.90	3.08	2.95	0.126	0.088	0.010	0.299
Glucose, mmol/L	3.04	3.44	3.65	3.60	3.18	0.121	0.454	0.606	0.074
Calcium, mmol/L	2.44 ^a^	2.39 ^ab^	2.47 ^a^	2.27 ^ab^	1.87 ^b^	0.069	0.027	0.007	0.070
Phosphorus, mmol/L	2.08 ^ab^	2.02 ^ab^	2.11 ^a^	1.89 ^ab^	1.48 ^b^	0.075	0.042	0.010	0.085
The 90th day of pregnancy									
Total Protein, g/L	76.02	78.67	89.30	93.82	75.77	2.493	0.102	0.350	0.016
Alanine Aminotransferase, U/L	40.10	38.16	43.45	44.62	33.79	1.816	0.352	0.632	0.159
Aspartate Aminotransferase, U/L	31.61 ^ab^	26.95 ^b^	40.04 ^ab^	47.49 ^a^	26.15 ^b^	2.531	0.018	0.520	0.037
Alkaline Phosphatase, U/L	34.51	45.10	50.57	64.66	38.29	4.452	0.229	0.379	0.082
Lactic dehydrogenase, U/L	364.14	210.02	359.02	407.50	219.12	28.860	0.077	0.618	0.444
Albumin, g/L	27.58	30.81	34.30	36.33	30.61	1.474	0.385	0.276	0.129
Urea, mmol/L	2.56	3.04	3.35	3.00	2.83	0.128	0.418	0.596	0.082
Glucose, mmol/L	3.40	3.03	3.16	3.72	2.74	0.177	0.499	0.621	0.606
Calcium, mmol/L	2.13	2.02	2.35	2.70	2.43	0.108	0.302	0.096	0.742
Phosphorus, mmol/L	1.94 ^a^	1.50 ^b^	1.79 ^ab^	1.94 ^a^	1.81 ^ab^	0.048	0.011	0.517	0.160

Note: In the same row, values with no letter or the same letter superscripts mean no significant difference (*p* > 0.05), while with different small letter superscripts mean significant difference (*p* < 0.05).

**Table 6:** Effects of FSHS on plasma hormone content of pregnant sows.

Items	Groups						*p*-Value		
	FSHS0 (Control)	FSHS2	FSHS4	FSHS6	FSHS8	SEM	ANOVA	Linear	Quadratic
The 60th day of pregnancy									
Insulin, μIU/mL	37.98 ^ab^	37.97 ^ab^	34.16 ^b^	43.24 ^ab^	89.14 ^a^	6.645	0.016	0.016	0.043
Progesterone, ng/mL	110.35	285.91	39.44	152.21	230.89	43.142	0.416	0.727	0.650
Estradiol, g/mL	67.59	64.05	75.02	90.24	40.38	8.047	0.415	0.624	0.200
Cortisol, ng/mL	62.24	53.08	71.12	58.48	69.01	3.333	0.428	0.430	0.758
Epinephrine, ng/L	64.66	70.96	53.39	74.46	60.65	3.767	0.436	0.867	0.961
Growth hormone, ng/mL	0.82	0.63	0.67	0.61	0.91	0.062	0.493	0.718	0.104
Insulin-like growth factor I, ug/L	20.48	23.38	23.65	23.11	30.01	2.312	0.787	0.281	0.725
Leptin, ng/L	2323.71	2442.47	1899.06	2170.18	2858.62	247.636	0.824	0.667	0.376
Ghrelin, ng/L	1717.41	1739.54	1547.41	1814.26	1665.74	34.355	0.148	0.902	0.669
The 90th day of pregnancy									
Insulin, μIU/mL	80.95	112.48	50.57	78.39	75.65	8.900	0.309	0.476	0.774
Progesterone, ng/mL	96.00	218.97	112.48	265.82	440.78	62.011	0.426	0.108	0.491
Estradiol, g/mL	430.42	371.39	525.99	277.24	505.82	42.097	0.338	0.848	0.625
Cortisol, ng/mL	67.27	73.29	66.53	54.54	79.44	3.336	0.184	0.805	0.231
Epinephrine, ng/L	68.34 ^a^	39.42 ^b^	53.07 ^ab^	40.77 ^ab^	54.01 ^ab^	3.518	0.045	0.220	0.033
Growth hormone, ng/mL	0.59	1.42	0.72	0.77	1.20	0.176	0.559	0.658	0.981
Insulin-like growth factor I, ug/L	19.11	39.42	53.07	40.77	54.01	0.970	0.716	0.645	0.402
Leptin, ng/L	2580.97	2704.32	2449.29	2187.99	2696.63	164.854	0.875	0.821	0.608
Ghrelin, ng/L	1698.11	1557.22	1634.44	1583.56	1653.00	19.121	0.134	0.615	0.062

Note: In the same row, values with no letter or the same letter superscripts mean no significant difference (*p* > 0.05), while with different small letter superscripts mean significant difference (*p* < 0.05).

**Table 7:** Effects of FSHS on plasma immunoglobulin content of pregnant sows (*n* = 30).

Items	Groups						*p*-Value		
	FSHS0 (Control)	FSHS2	FSHS4	FSHS6	FSHS8	SEM	ANOVA	Linear	Quadratic
The 60th day of pregnancy									
IgA	465.33	493.06	465.03	462.30	474.88	4.655	0.207	0.715	0.895
IgG	11.81	11.75	12.53	11.90	12.86	0.152	0.063	0.029	0.596
IgM	14.81 ^b^	15.88 ^ab^	16.04 ^ab^	15.28 ^ab^	16.30 ^a^	0.174	0.036	0.041	0.406
The 90th day of pregnancy									
IgA	528.73	503.82	528.18	517.45	529.09	8.710	0.887	0.829	0.630
IgG	12.92	13.19	13.87	13.19	13.19	0.149	0.368	0.488	0.143
IgM	16.29	16.95	17.38	16.89	17.01	0.287	0.853	0.525	0.446

Note: In the same row, values with no letter or the same letter superscripts mean no significant difference (*p* > 0.05), while with different small letter superscripts mean significant difference (*p* < 0.05).

**Table 8:** Alpha diversity of microflora in sow feces.

Items	Groups						*p*-Value		
	FSHS0 (Control)	FSHS2	FSHS4	FSHS6	FSHS8	SEM	ANOVA	Linear	Quadratic
The 60th day of pregnancy									
Shannon	4.10	3.81	4.05	4.30	4.97	0.600	0.411	0.251	0.125
Chao1	718.06	750.69	776.31	765.67	810.83	54.714	0.566	0.940	0.132
ACE	727.66	740.66	770.07	770.76	812.91	53.448	0.575	0.838	0.124
The 90th day of pregnancy									
Shannon	4.32	3.28	4.06	3.65	3.90	0.627	0.553	0.420	0.755
Chao1	696.98	639.87	675.27	601.12	663.70	72.673	0.731	0.515	0.532
ACE	699.39	645.51	682.24	608.80	668.80	72.348	0.755	0.553	0.559
During in-sow delivery									
Shannon	5.43	5.19	4.19	4.26	4.92	0.851	0.531	0.233	0.332
Chao1	756.72 ^ab^	912.66 ^a^	707.55 ^b^	756.77 ^ab^	910.87 ^a^	66.574	0.031	0.185	0.330
ACE	764.84 ^ab^	926.09 ^a^	713.41 ^b^	763.74 ^ab^	910.71 ^a^	67.143	0.032	0.216	0.409

Note: In the same row, values with no letter or the same letter superscripts mean no significant difference (*p* > 0.05), while with different small letter superscripts mean significant difference (*p* < 0.05).

**Table 9:** Abundance of fecal flora in sows at phyla level.

Items	Groups						*p*-Value		
	FSHS0 (Control)	FSHS2	FSHS4	FSHS6	FSHS8	SEM	ANOVA	Linear	Quadratic
The 60th day of pregnancy									
*Proteobacteria*	55.56	55.22	57.52	51.61	40.03	9.950	0.454	0.150	0.271
*Firmicutes*	29.61	27.01	26.03	32.98	35.00	4.024	0.204	0.092	0.138
*Bacteroides*	9.23	14.49	11.64	10.52	11.62	2.767	0.459	0.899	0.389
*Euryarchaea*	0.86	0.62	1.44	0.95	7.80	3.235	0.205	0.078	0.163
*Unidentified bacteria*	3.30	1.67	1.63	2.38	2.90	0.891	0.312	0.959	0.057
*spironema*	0.29	0.20	0.76	0.70	1.52	0.792	0.516	0.127	0.576
*Actinomyces*	0.20	0.29	0.17	0.10	0.26	0.080	0.226	0.762	0.400
*desulphurobacteria*	0.11	0.04	0.10	0.11	0.13	0.057	0.621	0.418	0.448
*Verrucobacteria*	0.14	0.04	0.13	0.07	0.03	0.047	0.141	0.108	0.702
*campylobacter*	0.03	0.01	0.07	0.02	0.04	0.033	0.384	0.718	0.837
The 90th day of pregnancy									
*Proteobacteria*	51.09	52.39	50.67	53.66	49.21	10.532	0.994	0.918	0.812
*Firmicutes*	15.96	28.42	16.35	21.71	17.89	6.402	0.329	0.846	0.393
*Bacteroides*	26.77	16.55	28.97	20.59	24.62	4.364	0.101	0.978	0.521
*Euryarchaea*	1.69	0.59	0.67	1.43	5.25	2.892	0.511	0.246	0.200
*Unidentified bacteria*	2.62	1.03	1.81	1.82	2.15	0.956	0.586	0.946	0.25
*spironema*	1.05	0.36	0.53	0.19	0.20	0.586	0.588	0.185	0.568
*Actinomyces*	0.12	0.06	0.13	0.08	0.03	0.056	0.440	0.230	0.488
*desulphurobacteria*	0.12	0.08	0.07	0.11	0.21	0.050	0.107	0.084	0.030
*Verrucobacteria*	0.05	0.01	0.06	0.02	0.02	0.024	0.249	0.241	0.950
*campylobacter*	0.03	0.00	0.02	0.00	0.01	0.020	0.452	0.221	0.479
Sow delivery									
*Proteobacteria*	23.89	33.02	52.04	44.43	40.45	20.486	0.701	0.354	0.353
*Firmicutes*	34.15	22.97	10.75	10.28	12.09	10.857	0.199	0.041	0.218
*Bacteroides*	32.39	31.11	27.43	28.57	33.96	8.201	0.924	0.975	0.422
*Euryarchaea*	4.96	4.55	5.12	11.75	8.05	5.899	0.713	0.334	0.973
*Unidentified bacteria*	2.57	5.44	2.61	2.66	2.93	1.205	0.154	0.464	0.484
*spironema*	1.26	0.61	0.84	1.32	0.83	0.906	0.918	0.942	0.813
*Actinomyces*	0.15	0.40	0.39	0.38	0.56	0.112	0.050	0.009	0.623
*desulphurobacteria*	0.02	0.12	0.10	0.11	0.10	0.050	0.359	0.218	0.186
*Verrucobacteria*	0.01	0.06	0.04	0.03	0.07	0.030	0.322	0.146	0.918
*campylobacter*	0.04	0.05	0.07	0.01	0.03	0.028	0.346	0.343	0.516

**Table 10:** Abundance of fecal flora in sows at the generic level.

Items	Groups						*p*-Value		
	FSHS0 (Control)	FSHS2	FSHS4	FSHS6	FSHS8	SEM	ANOVA	Linear	Quadratic
The 60th day of pregnancy									
*Herbaceous spirillum*	54.49	53.94	56.05	50.45	37.62	10.396	0.435	0.140	0.268
*Bacillus methanoides*	0.86	0.62	1.44	0.93	7.78	3.234	0.206	0.079	0.163
*Bacillus*	5.16 ^ab^	11.98 ^a^	4.53 ^ab^	5.03 ^ab^	4.23 ^b^	2.325	0.036	0.122	0.263
*Bacillus earthen*	2.73 ^c^	6.38 ^ab^	3.63 ^bc^	7.16 ^a^	2.04 ^c^	0.838	<0.001	0.752	<0.001
*Ruminococcus*	3.19 ^b^	1.69 ^b^	2.31 ^b^	2.77 ^b^	6.07 ^a^	0.522	<0.001	<0.001	<0.001
*Kristensenko, R-7*	0.92	0.66	0.97	1.49	2.50	1.011	0.430	0.108	0.329
*Streptococcus*	1.36	1.07	2.08	0.91	2.26	1.003	0.593	0.479	0.688
*Glycomonospora*	2.17	0.30	0.58	1.36	0.43	0.821	0.197	0.216	0.298
*Treponemas*	0.29	0.20	0.74	0.70	1.50	0.777	0.514	0.126	0.573
*Shigella Escheri*	0.43	0.21	0.73	0.38	1.54	0.581	0.236	0.095	0.247
The 90th day of pregnancy									
*Herbaceous spirillum*	80.95	112.48	50.57	78.39	75.65	8.900	0.309	0.476	0.774
*Bacillus methanoides*	49.19	50.98	49.67	52.50	48.09	10.686	0.994	0.978	0.775
*Bacillus*	7.08	26.03	13.43	19.02	11.94	5.820	0.066	0.838	0.052
*Bacillus earthen*	1.68	0.59	0.66	1.43	5.24	2.893	0.511	0.247	0.199
*Ruminococcus*	1.54	0.92	0.79	0.55	3.39	1.581	0.424	0.367	0.134
*Kristensenko, R-7*	2.73	1.84	3.68	1.28	1.41	1.394	0.435	0.329	0.570
*Streptococcus*	2.84	2.68	3.04	1.64	3.45	1.121	0.598	0.941	0.483
*Glycomonospora*	1.49	0.17	0.54	0.58	1.36	0.952	0.60	0.950	0.154
*Treponemas*	1.68	0.54	0.72	0.64	0.77	0.760	0.584	0.334	0.281
*Shigella Escheri*	1.10	1.16	0.61	1.85	0.70	0.834	0.614	0.952	0.778
Sow delivery									
*Herbaceous spirillum*	23.22	31.37	50.87	42.49	38.21	19.947	0.697	0.379	0.341
*Bacillus methanoides*	19.51	2.77	2.73	3.42	3.70	7.425	0.179	0.092	0.107
*Bacillus*	4.96	4.55	5.12	11.75	8.05	5.899	0.713	0.335	0.973
*Bacillus earthen*	1.73	9.00	1.37	0.72	2.36	4.745	0.443	0.524	0.740
*Ruminococcus*	6.94	0.78	1.41	4.36	3.14	3.419	0.433	0.611	0.207
*Kristensenko, R-7*	5.37	1.05	1.08	0.56	1.63	1.876	0.146	0.087	0.066
*Streptococcus*	1.87	3.48	4.97	2.54	3.98	2.128	0.641	0.508	0.468
*Glycomonospora*	2.22	4.37	1.87	1.82	2.86	0.903	0.088	0.539	0.926
*Treponemas*	1.24	0.57	0.82	1.29	0.79	0.885	0.906	0.934	0.819
*Shigella Escheri*	1.46	1.12	1.32	1.43	1.19	0.844	0.992	0.906	0.958

Note: In the same row, values with no letter or the same letter superscripts mean no significant difference (*p* > 0.05), while with different small letter superscripts mean significant difference (*p* < 0.05).

## Data Availability

All relevant data are within the paper. The data are available from the corresponding author on reasonable request.

## Supplementary Materials

The following supporting information can be downloaded at: https://www.mdpi.com/article/10.3390/ani14233389/s1, Table S1: Hormone and immune indicators in the plasma detection method.
