# Supplementary material for: Effects of Fermented Soybean Hulls on Reproductive Performance, Blood Physiology and Immune Parameters Along with Fecal Microbiota in Sows
Source: Animals (Basel). 2024 Nov 25;14(23):3389. doi: 10.3390/ani14233389 (PMC11640145; doi:10.3390/ani14233389)
Supplement: Supplementary file 1 [file animals-14-03389-s001.zip › animals-3196598-supplementary.pdf]

Table S1. Hormone and immune indicators in the plasma detection method

Items	Manufacturer, Name and specifications
Total Protein, g/L	Zhong sheng Bei kong biotechnology Co., Ltd, Assay kit for Total Protein
Alanine Aminotransferase, U/L	Zhong sheng Bei kong biotechnology Co., Ltd, Assay kit for Alanine Aminotransferase
Aspartate Aminotransferase, U/L	Zhong sheng Bei kong biotechnology Co., Ltd, Assay kit for Aspartate Aminotransferase
Alkaline Phosphatase, U/L	Zhong sheng Bei kong biotechnology Co., Ltd, Assay kit for Alkaline Phosphatase
Lactic dehydrogenase, U/L	Zhong sheng Bei kong biotechnology Co., Ltd, Assay kit for Lactic dehydrogenase
Albumin, g/L	Zhong sheng Bei kong biotechnology Co., Ltd, Assay kit for Albumin
Urea, mmol/L	Zhong sheng Bei kong biotechnology Co., Ltd, Assay kit for Urea
Glucose, mmol/L	Zhong sheng Bei kong biotechnology Co., Ltd, Assay kit for Glucose
Calcium, mmol/L	Zhong sheng Bei kong biotechnology Co., Ltd, Assay kit for Calcium
Phosphorus, mmol/L	Zhong sheng Bei kong biotechnology Co., Ltd, Assay kit for Phosphorus
Insulin, μIU/mL	Shanghai Enzyme-linked Biotechnology Co., Ltd., ELISA kit for pig Insulin (96T)
Progesterone, ng/mL	Shanghai Enzyme-linked Biotechnology Co., Ltd., ELISA kit for pig Progesterone (96T)
Estradiol, g/mL	Shanghai Enzyme-linked Biotechnology Co., Ltd., ELISA kit for pig Estradiol (96T)
Cortisol, ng/mL	Shanghai Enzyme-linked Biotechnology Co., Ltd., ELISA kit for pig Cortisol (96T)
Epinephrine, ng/L	Shanghai Enzyme-linked Biotechnology Co., Ltd., ELISA kit for pig Epinephrine (96T)
Growth hormone, ng/mL	Shanghai Enzyme-linked Biotechnology Co., Ltd., ELISA kit for pig Growth hormone (96T)
Insulin-like growth factor I, ug/L	Shanghai Enzyme-linked Biotechnology Co., Ltd., ELISA kit for pig Insulin-like growth factor I (96T)
Leptin, ng/L	Shanghai Enzyme-linked Biotechnology Co., Ltd., ELISA kit for pig Leptin (96T)
Ghrelin, ng/L	Shanghai Enzyme-linked Biotechnology Co., Ltd., ELISA kit for pig Ghrelin (96T)
IgA	Shanghai Enzyme-linked Biotechnology Co., Ltd., ELISA kit for pig immunoglobulin A (IgA) (96T)
IgG	Shanghai Enzyme-linked Biotechnology Co., Ltd., ELISA kit for pig immunoglobulin G (IgG) (96T)
IgM	Shanghai Enzyme-linked Biotechnology Co., Ltd., ELISA kit for pig immunoglobulin M(IgM) (96T)
